# “Discharge doesn’t mean the end”: Exploring success in discharge to community self-management for young adults living with chronic pain: A qualitative study

**DOI:** 10.1080/24740527.2024.2346943

**Published:** 2024-04-26

**Authors:** Souraiya Kassam, Emi Wong, Marysa Thompson, Todd Tran, Rachael Bosma, Sarah Sheffe

**Affiliations:** a Temerty Faculty of Medicine, Department of Occupational Science & Occupational Therapy, University of Toronto, Toronto, Ontario, Canada; b Women’s College Hospital, Toronto Academic Pain Medicine Institute, Toronto, Ontario, Canada

**Keywords:** chronic pain, young adults, transitions in care, discharge, specialized chronic pain services, community-based care

## Abstract

**Background:**

Living with chronic pain as a young adult (YA) can impact the physical, emotional, social, cognitive, and role function domains of life. Once YAs receive care for their specialist chronic pain care they are expected to self-navigate a complex health care system to transition to community-based care (i.e. primary care). Inadequate discharge planning may increase the unique difficulties YAs face in self-management, which may lead to adverse health outcomes, suboptimal discharge, and a need to reaccess care.

**Aims:**

The purpose of this qualitative study is to explore how YAs with chronic pain define a successful discharge transition from a health service delivery model of specialized chronic pain services setting to self-management in a community setting (i.e. primary care) and contextual factors that promote discharge success.

**Methods:**

This qualitative study included young adults with chronic pain. Data were obtained through semistructured interviews, which were transcribed verbatim and analyzed using inductive content analysis.

**Results:**

Ten participants identified that successful discharge includes the following considerations: (1) acknowledging the tension between moving forward and looking back, (2) a collaborative discharge process, and (3) the need for ongoing, relevant resources and support.

**Conclusion:**

This study provides a deeper understanding of how YAs with chronic pain characterize success in the discharge transition from specialized chronic pain services to community self-management. Our findings highlight the importance of provider–patient collaboration during the discharge planning process to develop a patient-centered self-management plan that incorporates community resources tailored to the needs of the individual to promote an optimal discharge.

## Résumé

**Contexte :** Le fait de vivre avec la douleur chronique en tant que jeune adulte peut avoir des répercussions sur les domaines de la vie liés aux fonctions physique, émotionnelle, sociale, cognitive et aux rôles. Une fois que les jeunes adultes sont pris en charge par un spécialiste de la douleur chronique, on s’attend à ce qu'ils naviguent de manière autonome dans un système de santé complexe pour passer à des soins communautaires (c.-à-d. des soins primaires). Une planification inadéquate de la sortie de l'hôpital peut accroître les difficultés uniques auxquelles les jeunes adultes sont confrontés en matière d'auto-prise en charge, ce qui peut conduire à des résultats de santé défavorables, à une sortie sous-optimale et à la nécessité d'un nouvel accès aux soins.

**Objectifs :** Cette étude qualitative vise à explorer comment les jeunes adultes souffrant de douleur chronique définissent une transition réussie entre la sortie d'un modèle de prestation de services de santé spécialisés dans la douleur chronique et l'auto-prise en charge dans un cadre communautaire (c.-à-d. de soins primaires), ainsi que les facteurs contextuels qui favorisent le succès de cette transition.

**Méthodes :** Cette étude qualitative a porté sur de jeunes adultes souffrant de douleur chronique. Les données ont été obtenues par le biais d'entretiens semi-structurés, qui ont été transcrits intégralement et analysés à l'aide d'une analyse de contenu inductive.

**Résultats :** Dix participants ont indiqué qu'une sortie réussie incluait les aspects suivants : (1) la reconnaissance de la tension entre le fait d’aller de l'avant et celui de regarder en arrière, (2) un processus de sortie collaboratif, et (3) le besoin de ressources et de soutien continus et pertinents.

**Conclusions :** Cette étude permet de mieux comprendre comment les jeunes adultes souffrant de douleur chronique caractérisent le succès de la transition entre la sortie d'un établissement spécialisé dans la douleur chronique et l'auto-prise en charge dans la collectivité. Nos résultats soulignent l'importance de la collaboration entre le prestataire de soins et le patient au cours du processus de planification de la sortie afin d'élaborer un plan d'auto-prise en charge centré sur le patient, intégrant des ressources communautaires adaptées aux besoins individuels, afin de favoriser une sortie optimale.

## Introduction

Chronic pain, or pain that lasts more than 3 months,^1^ affects approximately one in every nine young adults worldwide.^2^ It is common for chronic pain that begins in childhood to continue into adulthood: more than half of children with specific pain conditions have pain persisting into adulthood.^2–4^ For example, children with headaches are 3.6 times more likely to experience headaches in adulthood and those with back pain in childhood have a 2.4 times higher risk of back pain in adulthood.^4^ Because young adulthood (ages 18–25) is a developmental period associated with societal milestones and several contextual life changes, young adults (YAs) are susceptible to negative adult outcomes if their chronic pain is poorly managed.^5^ This can include negative impacts on academic achievement, physical activity, and social relationships, which can all have lasting effects on YAs’ lifelong socioeconomic status and functional well-being and mental health.^5–7^

Transitions of care can be especially stressful for YAs with chronic pain because they are newly expected to self-manage their condition and self-navigate through a complex health care system.^8^ For YAs with chronic diseases, health care transitions have been described as complicated, inconsistent, and inadequate.^9^ There is now a considerable body of research investigating the pediatric to adult health care transition for YAs with chronic pain.^10^ Factors that contribute to successful transitions from pediatric to adult specialist chronic pain care can include a collaborative approach between both service providers and YAs with chronic pain, adequate social support, and respect for YAs’ autonomy.^11^ However, in adult specialist chronic pain care services that are consultation based and offer time-limited, short-term care (the prevailing model in Canada), YAs who make the transition from pediatric to adult specialist care must also go through an additional care transition when being discharged from specialist care to the community care settings. Though attention to the posttransfer stage of pediatric to adult transition is strongly recommended, how to facilitate successful transitions beyond specialist care remains unclear, and little is known about whether adult pain care is promoting successful treatment of YAs following the initial transfer from pediatric pain care.^10^

According to research on chronic disease management, key contributors to a successful discharge from specialist care include communication between primary care providers and specialists with a clear summary and detailed care plan.^12–14^ When such factors are not in place, patients are less likely to adhere to treatment recommendations or have sufficient self-care skills and are more likely to experience readmissions, leading to increased health care utilization, costs, and long wait lists for specialist services.^15,16^ Moreover, very little is known about how to support YAs with chronic pain to achieve a successful discharge from specialist care.^10^ Therefore, the aims of this study were to explore how YAs with chronic pain characterize a successful discharge from specialized chronic pain services and to investigate the contextual factors that support discharge success. A qualitative methodology was chosen to elicit participants’ firsthand experiences and inform client-centered care. Findings from this practice-based study are key to informing clinicians working on interprofessional pain teams about how to support and facilitate successful discharge from specialist care settings.

## Methods

### Study Design

We conducted a descriptive qualitative study using semistructured individual interviews to explore the perspectives of YAs with chronic pain who have navigated the transition from adult specialist care to community-based care. This study was approved, and all methods were performed in accordance with guidelines and regulations by the research ethics board at Women’s College Hospital (REB No. 2021-0152-E) Toronto, Ontario, Canada and the University of Toronto (REB No. 42428), Toronto, Ontario, Canada.

### Research Team

Our research team included three master of science in occupational therapy students (S.K., E.W., and M.T.), two occupational therapists (S.S., T.T.) and a scientist from the Women’s College Hospital academic pain program (R.B.).

### Recruitment

A convenience sample of participants was recruited through health care providers from Women’s College Hospital, Toronto Academic Pain Medicine Institute and through social media advertisements. Health care providers contacted potentially eligible patients within their circle of care to inform them of the study. Additionally, study advertisements were shared via social media and potential participants who expressed interest in the study were asked to contact the research team by leaving a confidential message via voicemail or e-mail. All interested participants were contacted by the research team and were screened for eligibility. Participants were eligible to participate in this study if they self-identified as YAs (aged 18–28, inclusive), had been diagnosed with chronic pain (pain >3 months in duration), were currently or previously have received care from specialist chronic pain care service in Canada, and were able to communicate in English. The definition of “young adult” is highly variable in the literature, at times identified as 18–24 or 18–29.^2,10,17^ A range of 18 to 28 years was chosen for this study to include YAs with a 10-year range of ages who would have had recency in their adult health care or discharge experiences. Eligible participants were provided the study information letter prior to the interview date, and verbal consent was obtained from all interested participants who met the eligibility criteria.

### Data Collection

Qualitative data were collected using semistructured individual interviews that were completed via telephone or video conferencing from April to June 2023 (see supplemental material for interview guide). The team developed a 10-question interview guide to use with participants, and pilot testing of the interview guide was conducted. An online patient information and demographics survey was administered through a secure electronic data capture system (REDCap©) to be completed prior to each interview. The survey collected age, gender, education, ethnicity, pain diagnosis, and age of pain onset. The interviews were approximately 60 mins long and were conducted by M.T., a female occupational therapist student researcher with no prior relationship to the study participants. Information about the research team and goals of the study was shared prior to the interview. The interviews were modified to provide breaks and pacing according with the participants’ needs and were audio-recorded, transcribed verbatim, and reviewed for accuracy by the interviewer. Field notes were taken during and after each interview, where the interviewers documented a summary of participants’ verbal and nonverbal responses and their own impressions, observations, thoughts, and significant emotions from the interviews. All data were collected, managed, and kept securely according to REB guidelines.

### Data Analysis

We analyzed the participants’ interview transcripts using an inductive content analysis approach, as outlined by Elo and Kyngäs.^18^ NVivo 12 software was used to support coding and data storage. An inductive content analysis approach was selected because this methodology is particularly helpful when the goal is to describe and understand a phenomenon and derive practical suggestions or recommendations from the data.^18–20^ All transcripts were reviewed by team members. Two transcripts were independently re-reviewed by the research team (S.K., E.W., R.B., and S.S.) and codes were suggested that described key concepts from the interview. The team reviewed the suggested codes and co-created a preliminary coding list by grouping similar codes to capture as many aspects of the content as possible. Coding saturation was determined to be reached when new codes were not added from the narrative of successive interviews and sufficient context was established to describe the perspectives of YAs with pain related to discharge from specialist care. Once code saturation was reached in principle, the identified codes were applied by S.K. and E.W. as they coded the remaining transcripts independently. The code list was continuously refined through frequent (bi-weekly) review of the data by the research team. After the students completed the coding process for all transcripts, the research team (S.K., E.W., R.B., and S.S.) reviewed the master code book and engaged in the grouping phase, whereby codes were collapsed into categories. Lastly, the team (S.K., E.W., R.B., S.S., and T.T.) entered the abstraction phase of the inductive content analysis. Through collaborative discussion, the research team re-reviewed the data, code list, and categories and described themes. Together, the research team discussed different approaches to understanding and describing the themes that represented the data. When differences of interpretation arose, the team achieved consensus through discussion and reexamination of the data.

### Rigor

Core quality indicators of rigor, as described by Lincoln and Guba,^21^ include credibility, transferability, dependability, and confirmability.^21^ Emulating the rigorous approach described by Higginson et al.,^22^ we ensured credibility through a process of checking and rechecking across stages of data capture and analysis so that we accurately represent what was studied and provide supporting evidence by connecting our findings in the context of the previous literature.^22^ We provide detail on our participant sample and the study context such that our results can be interpreted and generalized appropriately to support transferability. Toward an aim of dependability, we describe the study methodology in rich detail, ensuring transparency in our approach to enable replication. To enhance confirmability, we engaged in reflexive practices throughout data collection and analysis, including reflexive dialogue between the research team members and shared field notes among team members during data analysis. Our team comprised experienced clinicians (12–25 years’ experience), researchers (>10 years’ experience), and trainees. Clinical members of our team work closely with this patient population, thus bringing expertise and potentially interpretation biases based on their past experience. As we interpreted the data, we reflexively questioned preconceived understandings and moved flexibly between raw data and content analysis to ensure that our interpretation aligned with the data and flexibly moving between raw data and content analysis.^23,24^

## Results

A total of 10 participants were included in the study: six women, three men, and one gender non-binary person. All participants contacted met the study criteria and there was no attrition. Participants’ ages ranged from 18 to 25 years old, with a median age of 22 years old. The age of pain onset ranged from 8 to 22 years old, with a median age of 14. Six participants were diagnosed with chronic primary pain, three with chronic secondary neuropathic pain, and one with endometriosis. See Table 1 for further details on participant information.

**Table 1.** Participant demographic information (*n* = 10).

Characteristic	Description
**Sex**	
*Male*	3 (30%)
*Female*	7 (70%)
**Gender**	
Cis*woman	6 (60%)
*Cis man*	3 (30%)
*Gender non-binary*	1 (10%)
**Age**	
*Median*	22
*Range*	18–25
**Ethnicity**	
*White (European decent)*	8 (80%)
*Black*	1 (10%)
*Latino/Latina/Latinx*	1 (10%)
*South Asian*	1 (10%)
*Indigenous*	2 (20%)
**Education**	
*Some high school*	2 (20%)
*Completed high school*	2 (20%)
*Some college/university*	3 (30%)
*College/university degree*	3 (30%)
**Main chronic pain diagnosis**	
*Primary pain*	6 (60%)
*Secondary neuropathic pain*	3 (30%)
*Endometriosis*	1 (10%)
**Age at pain onset**	
*Median*	14
*Range*	8–22

Three main themes were generated by the research team to describe how YAs with chronic pain define successful discharge from chronic pain specialty care and transition to community care and the factors, resources, and collaborative care required to support discharge success.

### Theme 1: The Tension between Readiness to Move Forward and Looking Back

Participants described how readiness for discharge from specialty care involves balancing a desire to return to “normal” (or pre-pain life) with the ability to accept their pain condition and to act on and maintain change.

Participants acknowledged the thoughts and emotions associated with change that may shape one’s readiness for discharge. This includes working toward acceptance of their pain condition and feeling increased confidence in the tools they have learned to manage the symptoms.

You know someone is ready when you can see them understand the knowledge that’s been given to them and not have a fear of themselves because there’s a fear with your body, especially with chronic pain. (Participant 5)

Participants frequently shared that YAs needed to change their mindset, emotions, and behaviors to become ready for discharge, but there were conflicting opinions about whether physical improvements or pain relief should be indicators for one’s readiness for discharge. Some participants felt that patients should not be discharged from specialist care until they had less pain. Other participants held a different perspective that pain and physical improvements were not indicators of discharge readiness because pain symptoms fluctuate significantly over time and may not reflect improved function.

Participants relayed how being ready for discharge was marked by engaging in enjoyable activities, increased independence, and a tangible difference in pain management behaviors. In this regard, a participant suggested that “when you […] do not give chronic pain the power to run your life,” YAs are ready for discharge (Participant 2). Conversely, most participants also indicated a desire for their lives to go back to “normal”—that is, to the way they were or would be without pain—and some participants suggested that being able to live a life like any other young adult would indicate readiness for discharge. Despite this paradox, participants agreed that patients who are ready to be discharged are patients who are living full, productive lives despite their pain.

You can tell when someone has had success, when you know they’re living their life and they’re doing the things that they need to do, they’re accomplishing the tasks that they need to do. (Participant 2)

### Theme 2: The Discharge Process Must Be Collaborative

This theme illuminates the importance of a collaborative discharge process between the client and health care professional. Shared decision making was one collaboration strategy promoted, and participants noted that to foster a truly collaborative therapeutic dynamic, YAs should play an active role in the decision-making process during discharge planning:

A successful discharge is when the patient and the provider both have […] a mutual agreement [that] it’s time to move on. (Participant 5)

Participants emphasized that discharge transitions should be seen as a process and recommended that there should be at least one appointment about the discharge process so that patients are aware and are prepared. Others affirmed that a rushed or abrupt discharge process would be detrimental to the transition in care process because it does not enable a focused discussion on how to continue managing pain postdischarge.

Appointments specifically for going over the discharge and going over things to do postdischarge … [to ensure that people are] empowered and knowledgeable enough to continue […] practices on their own. (Participant 10)

Participants also outlined that it is important that YAs feel confident, empowered, and knowledgeable about their pain management plan and have a clear understanding of how to access community supports and pain care if required in the future. This includes providing information on where to look for answers. In addition, participants noted that having a plan in place in case their pain escalated or evolved would help them be prepared to self-manage through the discharge transition. Participants described a person ready for discharge from care as being willing to take on responsibility for managing their own pain and feeling in charge of their plan. Conversely, someone who “isn’t, like, fully sure on everything they need to do to manage their condition […] probably shouldn’t be transitioned out because they’re being set for failure” (Participant 6).

Some participants described how uncertainty related to managing a chronic condition with episodes of increased pain symptoms complicates discharge from care, stating,

Who’s to say that in two years, it’s not going to flare up? I don’t … I think it’s hard to decide when to discharge somebody because their conditions aren’t linear. (Participant 11)

### Theme 3: Ongoing, Relevant Resources and Support Following Discharge Are Needed

A common response shared among participants was that a successful discharge would involve continued access to relevant resources and support once discharged. This included resources that were relevant to both their chronic pain needs and maximizing their prior knowledge. For example, one participant noted that

For it to be helpful, it needs to be relevant to the person. […] if I’m being sent to a program that’s all about, you know, walking […] I literally physically cannot do it because my joints, you know, lock up and stiffen and hurt. That’s not going to be helpful. (Participant 6)

The need for these resources to be tailored to the individual needs of each client was emphasized. Identifying resources that were accessible included considering health literacy, comfort/accessibility of technology to access the resources if necessary, and affordability. Affordability was raised by many participants because pain services in the community, such as physiotherapy or counseling, are not often covered by public health insurance. When suggested resources for self-management were too expensive, such as private therapies, YAs felt like the health care provider is giving “hope, but [the patient] can’t reach it” (Participant 5). As such, affordability can be a barrier to client-centered care during the discharge transition, which may increase the difficulty YAs experience in self-managing their chronic pain.

Once discharged with appropriate, patient-tailored resources, participants shared that their ability to manage their chronic pain optimally can also be influenced by their access to ongoing pain support. Supports recommended by participants included being connected to a health care provider with whom they could occasionally check in about pain management; for example,

[Having] appointments periodically to kind of make sure that plans for keeping […] on track again … are being adhered to. (Participant 3)

Many participants expressed concern about access to support in the event of pain worsening in the future postdischarge and indicated that having a contact they could reach out to would help them feel less alone in a transition out of routine specialist care.

Participants commonly reported an understanding that “discharge doesn’t necessarily mean the end […] of treatment” (Participant 10). Instead, discharge marked a transition to a period of less routine access to health care. Though ongoing care and support may look different postdischarge, participants noted that health care providers still played an important role in facilitating a successful transition to self-management and would be desired on an ongoing, albeit less frequent, basis upon discharge to the community. Some participants also pointed to positive emotional aspects of decreased frequency of contact with health care providers. For example, Participant 1 stated,

There’s, like, a proud feeling of, like, getting to a place where you don’t really need that [… support] getting to a point where, like, you’re good on your own two feet.

Social support was also identified by participants as vital to help continue progressing toward their goals once discharged into the community. This included partners, family members, and friends who could serve as a “teammate” (Participant 6) in carrying out their pain management plan. Some participants noted that having social support was especially important during health care transitions to foster accountability and help with pain management.

I think having somebody who just, like, even if they ask me what I want to do, and I’m the one who’s supplying the options, […] having somebody who is aware of [my plan] can help me be accountable to executing it. (Participant 1)

## Discussion

The purpose of this study was to explore how YAs with chronic pain characterize a successful discharge from specialized chronic pain services and to investigate the contextual factors that support discharge success, elucidating how and when to transition patients from specialist to community care settings. Key themes identified through this study were the tension between moving forward and looking back, the discharge process must be collaborative, and ongoing, relevant resources and support following discharge are needed. Participants identified experiences of being discharged from specialty chronic pain care and provided insights on how the transition process could enable more successful discharge to support their pain management in the community.

The findings indicate that YAs with chronic pain experienced fear that discharge into the community would be a sudden, one-time event that occurs abruptly at the discretion of health care providers. From a health service delivery perspective, this is consistent with research exploring the discharge experiences of adult patients with one or more chronic illnesses from the hospital (e.g., specialist care) to community (e.g., primary care, etc.).^25,26^ Previous studies indicated that a lack of communication between health care providers and patients can make discharge from care feel abrupt and overwhelming.^25,26^ YAs with chronic pain emphasized the need for discharge planning to be a collaborative process instead, in which the patient has a partnership with the health care provider in creating their discharge plan.^27,28^ Our findings align with other literature showing that engaging patients more collaboratively (e.g., through discussion, answering questions, addressing concerns) during the discharge process can lead to an increase in perceived readiness for discharge similar to adult patients being discharged home from inpatient rehabilitation services.^29^ Our study highlighted the perceived need identified by patients to be more collaboratively engaged in discussions around discharge, and Knier et al. tested a discharge intervention that included more patient engagement and found that it improved overall patient satisfaction.^29^ Furthermore, our study builds on the chronic pain literature by presenting evidence to support the importance of a collaborative discharge process specifically for YAs from specialty chronic pain services. Though patient collaboration is ideal for an optimal discharge experience, this may be challenging for chronic pain clinics to achieve. Previous literature on outpatient settings has identified the lack of time of health care providers and clinic organization as system barriers to collaborative discharge planning.^26^ For example, patients have reported that their time with health care providers can feel too rushed to ask questions or too limited for their health care provider to discuss patient concerns, because appointments are usually quick consultations.^26^ Communication between specialist care providers and primary care providers in the community also remains challenging and does not consistently occur, thus impeding smooth transitions in care between care settings.^30,31^ Further exploration of how systemic constraints can be overcome to facilitate successful discharge transitions for YAs with chronic pain is needed.

As age increases, the number of unplanned hospital readmissions has been found to increase significantly for young adults with chronic diseases, highlighting the critical need for discharge care planning.^32^ Consistent with findings related to discharge success in other diseases and other care setting transitions (e.g., hospital to home), our study participants indicated that they require a clear understanding of future care pathways and accessible resources to support pain management after discharge.^26,33,34^ Though there are no defined protocols for discharge from specialist pain care, YAs with chronic pain indicated that they were better equipped for self-management when they were given resources tailored to their unique needs. For example, participants emphasized the need for resources that were affordable and that considered their individual knowledge, interests, and goals. This is a clear indication of the necessity of client-centered care among physicians and interprofessional health care disciplines. Our present study indicates that YAs with chronic pain feel more empowered to manage their pain on their own when they have a clear pain management plan upon discharge.

Some participants reported that their sense of readiness can significantly impact their experiences of being discharged and should therefore be considered by health care providers during the discharge process. Shaikh and Hapidou explored factors that determine a patient’s perception of self-improvement after participating in a chronic pain program.^35^ The authors found that increased readiness for change was associated with significant changes in self-improvement. Additionally, research by Kerns and Rosenberg explored the application of the transtheoretical model of change as a predictor of patient responses with individuals with chronic pain.^36^ Kerns and Rosenberg determined that readiness for change was effective in determining adult patients’ willingness to engage in self-management treatment.^36^ YAs in our study identified a similar construct that their own readiness for change was also a key factor to being ready to be discharged and discharge success, which further contributes to the chronic pain literature from a YA perspective of living with chronic pain. Though physical symptom management or cutoffs have been used as indicators for discharge readiness in other chronic diseases (e.g., chronic obstructive pulmonary disease), the YAs in our study were conflicted as to whether their level of pain or associated symptoms should be a factor in their discharge planning.^37^ Because pain perception is subjective and may fluctuate in intensity or duration, pain indicators, such as intensity, may be unsuitable as discharge readiness indicators. Instead, participants indicated that functional gains should be the focus. Our findings suggest that health care providers should be cognizant of patient readiness across multiple domains (e.g., symptomatically, emotionally, behaviorally) as one of the contextual factors to optimize the success of discharge planning. Tools such as the Transition Readiness Assessment Questionnaire may be useful in identifying readiness and facilitating effective planning.^38,39^ In preparation for discharge, pain specialists should work collaboratively with patients to co-create care plans that outline pain management strategies (e.g., medications, therapies, resources, what to do if pain worsens) that are focused on improving function or adaptive behavior, work to ensure that YAs learn the skills and adaptive behaviors required to enact this care plan, and confirm that YAs understand how to find and access community resources through the discharge transition.

Of note, participants highlighted that it is important to consider the emotional and social elements associated with the discharge process. For example, participants reported that the discharge process may be associated with (sometimes mixed) feelings of fear, acceptance, or confidence.^40^ In particular, our findings exemplified that a sense of confidence was described as a key emotional element for discharge success. Furthermore, participants highlighted the need for social support and named support from peers, friends, and family members as key to their pain management success. This aligns with the biopsychosocial model of pain care and highlights the well-known importance of social support for pain management.^41^ Health care providers can work with YAs to cultivate the social support that they require and can assess and discuss YAs’ confidence in their knowledge of their pain management plan, ability to use ongoing self-management strategies, and knowledge of how to access care in the future if needed. Addressing YAs’ needs and concerns in these areas may decrease fear around discharge and contribute to discharge success.

## Strengths and Limitations

Social positionality can significantly influence individuals’ experiences with chronic pain.^40^ Thus, the inclusion of participants from certain underrepresented groups in chronic pain research is a strength of this study, because it explores the experiences of discharge from diverse YA chronic pain service users that are often unheard. Future research can build on this to further explore the experiences of other underrepresented groups, including those not represented in this study. Additionally, our study recognizes that there is not a singular journey through health care, and we do not know whether our participants have had other experiences with discharge that could influence their experience/perspective on factors and conditions that influence successful discharge. For example, some participants had not yet been discharged from specialist care whereas other participants had, and those who had reported variable success. Therefore, the generalizability of our findings can be limited. Another strength of this study is its contribution to the chronic pain literature from a YA perspective, specifically focused on experiences within adult health care. These perspectives are underrepresented because the pain literature tends to focus either on the pediatric to adult health care transition or on adults living with chronic pain rather than a younger population with their own unique experiences and challenges.

This study suggests several clinical implications for YAs living with chronic pain experiencing a transition process from specialty chronic pain services to community care. First, this research provides evidence to support the need for health care providers working in chronic pain specialist care settings to partner with YAs in their discharge planning process to ensure their readiness for discharge. This study also supports the need for the structure of discharge planning to include discussions of discharge in advance, to warrant enough time for YAs to develop and understand their self-management plan with the help of their health care provider, share concerns about discharge, and build confidence. Allotting more time to the transition process can also allow specialist health care providers to identify ongoing individual needs and how these may be met in the community. This time can also provide more tailored resources for patients to support their pain management, which this study identified is important to YAs with chronic pain. The investment required to conduct thorough and collaborative discharge planning requires upfront investment in health care resources with the goal of ultimately streamlining services downstream by reducing the need for re-referral or emergency care.

## Conclusion

Our present study explored how YAs with chronic pain characterize a successful discharge from specialized chronic pain services to community care to understand the contextual and personal conditions that support discharge success. Participants highlighted that discharge is a process, not a one-time event. Additionally, the findings of this study highlight the need for collaborative, interprofessional involvement from a multifaceted health care team and the importance of client-centered, individualized discharge planning with the YA population.

## Supplementary Material

Supplemental Material
