# Supplementary material for: “Discharge doesn’t mean the end”: Exploring success in discharge to community self-management for young adults living with chronic pain: A qualitative study
Source: Can J Pain. 2024 Apr 26;8(1):2346943. doi: 10.1080/24740527.2024.2346943 (PMC11633132; doi:10.1080/24740527.2024.2346943)
Supplement: Supplemental Material [file UCJP_A_2346943_SM0417.docx]

**Health care transition**

1. Tell me a little about yourself and your chronic pain?
2. When have you felt successful in managing your pain? What has helped you most to be successful in managing your pain?
   1. What kinds of supports from healthcare specifically have been the most beneficial to you? (and how have they been beneficial?)
3. If you were to describe another person as “successfully discharged” or “successfully transitioned” from a specialty pain clinic back to their primary care provider, what would they be **doing** that tells you they’re successful?
4. How would a person ready to be discharged from specialty pain clinic **describe** themselves? What would they be **doing** that tells them they are ready?
5. What would make this transition from being a patient to being discharged go well or go poorly?
6. How would having strategies and resources for self-management or community care would be helpful or unhelpful?
7. How would someone need to be supported for transition to managing without specialty health care services?
8. What do you know about that could help you or has helped you be successful in managing your pain outside of the specialty pain clinic?
9. If you have transitioned to/ been discharged to community, did you feel ready? What if anything would you have liked to be different?
10. Finally, is there anything else you would like to share about the experience of transition from specialty chronic pain care into self-management?
